# Dengue en Argentina: la confirmación de nuestras peores sospechas

**DOI:** 10.31053/1853.0605.v81.n1.44575

**Published:** 2024-03-27

**Authors:** Adrián Diaz

**Affiliations:** 1 Universidad Nacional de Córdoba. Facultad de Ciencias Médicas Instituto de Virología "Dr. J. M. Vanella"; 2 Consejo Nacional de Investigaciones Científicas y Técnicas. Investigador independiente

Mientras escribo esta editorial se desarrolla en nuestro país (Argentina) la peor epidemia de Dengue desde su reintroducción en 1997. Es la peor en magnitud de casos, por expansión geográfica y muy probablemente por duración en meses.

Sin embargo, esta no es una situación ajena para la región ni para el mundo. Según la Organización Panamericana de la Salud, en lo que va del año, se reportaron un total de 1.874.021 casos sospechosos de dengue, cifra que representa un incremento del 249% en comparación al mismo periodo del 2023 y 354% con respecto al promedio de los últimos 5 años^1^. A nivel mundial, la incidencia del dengue ha crecido dramáticamente en los últimos 30 años. Entre 2000 a 2019, el número de casos reportados a la Organización Mundial de la Salud aumentó 10, 28 veces (505.430 en 2000 a 5,2 millones en 2019)^2^. Algunas estimaciones indican que se producen 390 millones de infecciones anuales por el virus dengue (VDEN), de las cuales 96 millones se manifiestan clínicamente^3^. La enfermedad es ahora endémica en más de 100 países y se estima que 3,9 mil millones de personas están en riesgo de infectarse con el virus dengue. El 70% de los casos se reportan en Asia y la región de las Américas es una de las regiones más afectadas.

En lo que va de esta epidemia 2023-2024, se registraron en Argentina 120.007 casos de dengue en 19 jurisdicciones, de los cuales 248 casos fueron dengue grave (0,20 %) y 79 fallecidos (0,07%)^4^. Si observamos con atención la Figura 1 podemos ver que el inicio de casos se da aproximadamente 17 semanas epidemiológicas antes que el inicio de la última epidemia y para esta misma época del año se han registrado 9 veces más casos que el año anterior.

**Figura Nº 1:**
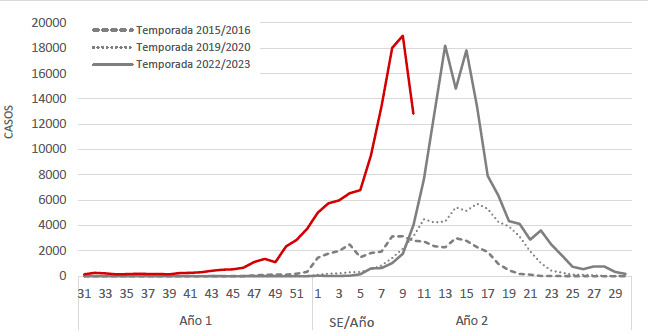
Evolución histórica de los casos de dengue detectados durante las mayores epidemias registradas en el país^4^.

En la región del noreste argentino la notificación de casos de dengue fue continua todo el año sin observarse un corte en la transmisión vectorial en el invierno. La tendencia histórica que observamos en Argentina nos hace pensar frente a un posible proceso de endemicidad del virus principalmente para las regiones subtropicales del país.

En Argentina, el vector del VDEN se reintroduce en 1986 en la provincia de Misiones y el virus lo hace en 1997 registrándose los primeros casos en la provincia de Salta. Desde ahí los casos han ido en aumento y las epidemias se han vuelto más frecuentes y afectando mayor número de personas (Figura 1). Es innegable que el calentamiento global en nuestro país está generando eventos extremos de temperaturas cálidas, aumento de la temperatura promedio y acortamiento de los inviernos. Esto tiene una repercusión directa sobre la dinámica de transmisión del VDEN. Al incrementarse las temperaturas promedio y acortarse el período invernal, la actividad de vuelo y de alimentación y potencial transmisión del VDEN por el mosquito se amplía a épocas del año donde antes no estaba activo. Además, con el incremento de la temperatura, se genera un acortamiento de los tiempos que necesita el mosquito para transmitir el VDEN (disminución del *período de incubación extrínseco*) por lo tanto podemos tener mayor número de ciclos de transmisión y mayor número de infectados en el mismo período de tiempo. Además, el incremento promedio de las temperaturas como el corrimiento de las áreas de precipitación ha hecho que la distribución geográfica del mosquito vector se amplíe a nuevas regiones de nuestro país. La distribución histórica del *Ae. aegypti* abarcaba desde el norte y centro de Argentina, siendo las provincias de Córdoba, Santa Fe y Buenos Aires los límites de distribución. En la actualidad, el mosquito se ha establecido en regiones impensadas como Cuyo (Mendoza, San Juan, La Rioja) y norte de Patagonia (La Pampa, Neuquén). El aumento de la zona urbanizada sin planificación es un factor importante a la hora de evaluar la problemática del dengue. Cientos de miles de personas se establecen en áreas urbanas periféricas sin contar con el servicio de red de agua, hecho que obliga a estas poblaciones a acumular agua en tanques y diversos reservorios que se convierten en sitios de cría para el mosquito. Los traslados de personas por diferentes motivos han incrementado el intercambio de serotipos de VDEN aumentando las probabilidades de la hiper-endemicidad del virus y del aumento de casos graves de dengue.

La problemática del Dengue, como toda problemática sanitaria, es atravesada por diferentes dimensiones que van más allá de la médica y la epidemiológica. Las dimensiones biológicas (conocer la biología del vector), sociales (condiciones de vida de las personas), culturales (hábitos comportamentales) y políticas (regulaciones, leyes, presupuestos en salud pública y control vectorial) deben ser consideradas también en la toma de decisiones. Esto requiere de una mirada y abordaje integral, de un diálogo constructivo entre todos los actores que componen la sociedad y las instituciones públicas para que de esta manera se delinee una estrategia integral sustentable y a largo plazo para disminuir el impacto del Dengue en nuestra población y región.
